# Supplementary material for: The streaming of plastic in the Mediterranean Sea
Source: Nat Commun. 2022 May 27;13:2981. doi: 10.1038/s41467-022-30572-5 (PMC9142569; doi:10.1038/s41467-022-30572-5)
Supplement: Supplementary file 1 — Supplementary Information [file 41467_2022_30572_MOESM1_ESM.pdf]

Supplementary Note for
The streaming of plastic in the Mediterranean Sea

Alberto Baudena[1*], Enrico Ser-Giacomi[2†], Isabel Jalón-Rojas[3†], François Galgani[4], and Maria Luiza Pedrotti[1]

[1]Sorbonne Université, CNRS, Laboratoire d'Océanographie de Villefranche, UMR 7093 LOV, Villefranche-sur-Mer, France
[2]Department of Earth, Atmospheric and Planetary Sciences, Massachusetts Institute of Technology, 54-1514 MIT, Cambridge, MA 02139, USA.
[3]CNRS, UMR5805 EPOC, University of Bordeaux, 33615 Pessac, France
[4]French Research Institute for Exploitation of the Sea (IFREMER), Bastia, France

* Correspondence to: alberto.baudena@gmail.com.

This pdf file includes:

Supplementary Note

Supplementary Figs. 1 to 19

Supplementary Table 1

Supplementary Note

1 *In situ* vs model comparison: sensitivity analysis

1.1 Plastic categories, horizontal diffusion K_h, and half-life on the beach T_W

Virtual particle concentrations in each Mediterranean sub-basin were compared to the eight categories of *in situ* concentrations of plastic debris (Material and Methods, Section 1.1). In Scenario M, significant correlations were found between the *in situ* plastic-debris concentrations and the model values in the following categories (Supplementary Fig. 4, first panel):

- **Category 2:** (particles of size 5–20 mm, g/km^2), R^2=0.91, p<0.05 (Pearson test);
- **Categories 3,4:** (particles of size greater than 20 mm and of all sizes, respectively, g/km^2), R^2=0.92 and R^2=0.96 respectively, p<0.01;
- **Categories 7,8:** (surface covered by plastic debris and by plastic fragments, respectively, m^2/km^2), R^2=0.93, R^2=0.96, respectively, p<0.01.

When *in situ* plastic-debris concentration was expressed as the number of items per km^2, no significant correlation was found (p>0.05). This could be due to the fact that the number of plastic items depends on fragmentation. This dynamic was not included in the present version of the model due to the lack of quantitative information on this process.

The correlations with the *in situ* concentration were calculated for each of the 16 scenarios separately. Significant correlations were recurrently found for the same plastic concentration categories (Supplementary Fig. 4, lower panel): Category 2 (except for the scenario with T_W=50 days K_h=15 m^2/s), and Categories 3, 4, 7, and 8. The persistence of the correlations across the different scenarios strengthens the decision to average together the outputs of the 16 scenarios.

1.2 Proportions of particles emitted from cities p_C, rivers p_R, and vessels p_V

The correlations with the *in situ* concentration were calculated using four different proportions of particles emitted from cities p_C, rivers p_R, and vessels p_V (upper table of Supplementary Fig. 5). Case 1 (40-40-20%) is usually assumed in the literature (*1*), Case 2 (62-32-6%) was recently estimated by (*2*) for the Mediterranean Sea, while Cases 3 and 4 represent intermediate proportions. Significantly correlations were recurrently found for the same plastic concentration categories (Supplementary Fig. 5A–D, Categories 2, 3, 4, 7, and 8), but the largest correlations were found with the proportions 50-30-20% (Supplementary Fig. 4, upper panel). These results indicate a robustness of the model with respect to these parameters, and corroborate the p_C, p_R, and p_V proportion used.

2 *In situ* vs model comparison: distance from shore

To assess the variability in particle concentration as a function of distance from shore, *in situ* concentrations at the different stations were regrouped according into four shore-distance classes: stations (i) less than 20 km (inner continental shelf); (ii) between 20 and 40 km (middle continental shelf); (iii) between 40 and 60 km (far continental shelf); and (iv) more than 60 km from the coastline. For each distance class, the mean concentration and standard error were obtained. In the model, the distances from the shore were calculated for each trajectory point, at each daily time step. The distances obtained in this way were then regrouped according to the distance classes specified above. Finally, the *in situ* and virtual concentrations were normalized to permit comparison.

For Scenario M, there was agreement between the simulated and observed concentrations for Categories 1 and 2 (Supplementary Fig. 6A–B; R^2=0.91 and R^2=0.94, respectively, $p<0.05$, Pearson test). Large particle concentrations were found close to the shore, with few particles in

a strip between 20 and 60 km, followed by an increased concentration in the open sea (> 60km). This result is consistent with previous measurements of plastic distribution as a distance from shore in the Mediterranean Sea (*3*). However, a larger observed concentration close to the coast could also be explained by a greater number of large debris (heavier) occurring close to land sources. To verify this hypothesis, we calculated the size distribution for each class and we normalised it, so that the sum of the abundance of a given class was equal to 1 (Supplementary Fig. 6C). The size distribution did not change considerably when changing the shore distance. A slight difference is present in class 40–60 km, likely due to the relatively number of in situ stations (7 out of 122). The results show that the distribution of plastic concentration as a distance from the shore is not explained by a greater occurrence of larger items close to the shore. Furthermore, they indicate the coastal area as a region of debris retention, in accordance with our results (e.g. Fig. 5, Supplementary Fig. 12).

3 Simulated plastic budget

The total number of particles that beached, sank, or that were on the surface (expressed as a percentage of the total number N of particles released between January 2013 and December 2016) was calculated for each month of 2013–2016 for Scenario M (Supplementary Fig. 7, upper panel). The result refers to the ensemble average obtained from 4 biofouling times (50, 100, 150, and 200 days; M 1.2.5 and 1.2.6), shown separately in the second panel of Supplementary Fig. 7.

The number of sinking and beached particles increased linearly with time, representing, at the end of December 2016, $\sim$86.5 and $\sim$11.5% of particles, respectively. Floating particles constituted the remaining $\sim$2.2%; this percentage oscillated, increasing during winter and decreasing during summer. This could be due to the fact that the number of particles released from the rivers was larger during the winter months or that storm frequency was maximal during winter;

this fostered both particle beaching and washing-off. Washing-off effect was dominant over beaching in winter: the number of beached particles increased more slowly then (red line). This increased the number of particles in the water during winter. However, we stress that these results are still uncertain, and should be considered carefully, as both the beaching and washing-off modeling had limitations.

Remarkably, the number of particles in the water became steady after just 4–5 months from the beginning of the release. It also did not change significantly when the biofouling time (second panel of Supplementary Fig. 7), the horizontal diffusion coefficient (K_h) and half-life of beached plastics (T_W) were varied (Supplementary Note S.4 and Fig. 8). The third panel of Supplementary Fig. 7 shows the mean time spent in the water by the particles according to their origin and to the biofouling time. Particles spent on average between 20 and 30 days in the water. This value was slightly lower for particles originating from terrestrial sources and consistently higher for plastics originating from vessels (40 to 90 days), in accordance with (*4*), but the values obtained here were slightly higher. This difference was likely due to the different description of washing-off and a greater T_W (one order of magnitude larger) in the present study, which favored a longer time spent by the particles in the water (M 1.2.4).

Despite the fact that 80% of particles were released from land sources, almost the half the sinking particles were originated from vessels (fourth panel of Supplementary Fig. 7). As the biofouling time increased, the number of sinking particles declined. The percentage of sinking particles with a biofouling time of 50 days (8% and 10% for vessel and land sources, respectively) was different from the values obtained by (*4*) (6% and 3.2%, respectively). Again, this could be related to the different washing-off dynamics used in the present study.

4 Sensitivity of the plastic budget

Supplementary Figure 8 shows the plastic budget for each of the 16 scenarios, analogous to that reported in Section 3 (percentage of particles that were floating, beached or sunk during the period of particle release, 2013–2016). The trends did not change significantly across the 16 different panels. The number of beached plastics decreased to about 5% when T_W was increased from 25 to 100 days, and when the horizontal diffusion K_h was decreased from 15 to 0 m^2/s. This decrease was offset by a corresponding increase in biofouled particles. The percentage of particles in the water, on average, did not seem to be affected by changes in T_W or K_h.

5 Validation of the advective time length

Here we discuss the decision to advect the virtual particles for a period of $\sim$380 days (the advective time). This represented a trade-off between an acceptable representation of the cycle of plastic particles at sea and the optimization of computational performances. As seen in Section S.3, the percentage of virtual particles yet not beached or sunk, that is particles still floating in the water at the end of the advective time, constituted only $\sim$2.2% of the particles released (upper panel of Supplementary Fig. 7). Such a value was consistent with the findings of (*4*). In addition, it was consistent with the short mean time spent in the water by the particles (Supplementary Fig. 7, third panel), which was much shorter than the advective time.

Of the particles that were still floating in the water at the end of the simulation (Fig. 9), the majority (80%) were found less than 40 km from land (Fig. 9, first panel). In addition, these particles were beached and washed off a consistent number of times and with a greater frequency than the other particles (Fig. 9, second and third panels). This suggests that the majority of these particles remained close to the shore, where they were continuously beached and resuspended. It is likely that this process continued until they became permanently beached or sank

due to biofouling. This coastal-retention dynamic has also been suggested by (*5*). For all these reasons, we considered that an advective time of $\sim$380 days permitted a realistic simulation of the plastic-debris cycle in the Mediterranean basin. Finally, we note that the advective times used in previous Mediterranean Lagrangian studies targeting plastic debris were either shorter or similar that adopted here (e.g., (*6–8*))

6 Sensitivity test of crossroadness and plastic crossroads

6.1 Horizontal diffusion K_h and half-life on the beach T_W

The crossroadness fields found for each of the 16 scenarios separately were almost identical (Fig. 10). The intensity slightly increased for higher T_W values, due to the fact that fewer particles remained on the beaches. Remarkably, the positions of the plastic crossroads were very similar across the different scenarios: the crossroad close to Mallorca island and the three crossroads in the Adriatic basin did not change position. Three or four crossroads were always present in the Cilician basin, three or four persisted in the Turkish coastal sector of the Aegean sea, and five or six along the Algerian-Tunisian shore. Finally, two crossroads were always found in proximity to the Bomba Gulf in Lybia, and a crossroad always close to the Suez Canal. The ranking of the crossroads varied slightly, but the percentage of intercepted particles as a function of the number of crossroads considered did not change significantly (not reported). This quantity increased slightly with increasing T_W (fewer particles retained on the shore) and with decreasing K_h (less dispersion). These considerations indicate a robustness of the plastic crossroads with respect to changes in the horizontal diffusion coefficient K_h and the half-life of plastics on the beach T_W.

6.2 Radius σ of the circular crossroads

The crossroadness sensitivity was tested against changes in the radius σ defining the extent of the crossroads. Two additional σ values were tried: 0.2° and 0.3°. In both cases, a buffer of 0.1° from land sources was imposed. A larger σ allowed the crossroads to intercept more virtual particles, thus increasing the crossroadness (Fig. 11). However, the two crossroadness patterns remained almost identical. Notably, in both cases the disposition of the most important crossroads matched that for σ=0.1°, the value chosen. The only exception was the emergence of two crossroads (for both 0.2° and 0.3° σ), the first in the open sea in the Gulf of Sidra, the second in the Ligurian-Provençal Sea, south of the Hyéres Islands. The only significant change occurred to the ranking of the crossroads, mainly because they covered a larger surface. These considerations illustrate that the distribution of the crossroads was robust to changes in the crossroad area.

With σ=0.1°, 60 crossroads were necessary to intercept $\sim$20% of the particles (Fig. 5), $\sim$30 crossroads with σ=0.2°, and $\sim$25 crossroads with σ=0.3° (not reported). The total surface area covered by these crossroads was 0.93%, 1.6% and 2.8%, respectively, of the Mediterranean surface. This highlights the capacity of the algorithm to decrease the total surface covered by the crossroads selection as σ decreases. Future higher-resolution studies could benefit from this aspect, improving the performance of the crossroads identification.

6.3 Buffer around land sources

Crossroad could not be located inside the buffers around land sources. Here, we calculated the percentage of particles that stayed inside these buffer regions while varying their size. On increasing the buffer size from 0.1° to 0.3°, the percentage of particles retained inside the buffers increased only slighlty, from $\sim$67% to $\sim$70% (Fig. 12); at a buffer size of 0.5°, it increased to $\sim$76%. The percentage of particles intercepted by the crossroads also did not change signif-

icantly when the size of the buffer region was increased. This indicates the soundness of the crossroadness analysis with respect to this parameter.

6.4 Proportions of particles emitted from cities p_C, rivers p_R, and vessels p_V

Plastic crossroadness and 20 most important crossroads were calculated using four different proportions of particles emitted from cities p_C, rivers p_R, and vessels p_V (upper table of Supplementary Fig. 5). The results (Fig. 13) show that the crossroadness slightly changed only in Case 2, due to the lower input from vessels, but the pattern remained identical. Neither did the crossroad disposition vary, but only the ranking. These results show the robustness of the crossroadness and the crossroads with respect to these parameters.

6.5 Biofouling time

Here we investigated the importance of biofouling in the crossroads identification by calculating the total time that particles had spent in the water when they were intercepted by a crossroad, and comparing it with the biofouling time. After a certain amount of time in the water, it was possible that the particles were sufficiently biofouled and sank, and would therefore not be intercepted by the crossroads. The upper panel of Fig. 14 shows the cumulative pdfs of the time spent in the water by the particles intercepted by each of the first 10 crossroads. Some crossroads, such as the 2nd or the 4th, mainly intercepted particles that had spent relatively short time in the water ($\sim$95% less than 50 days). For other crossroads, such as the 6th and the 8th, this percentage was lower ($\sim$50%). Overall, however, the cumulative pdfs were similar. On average, $\sim$78% (96)% of the particles had spent less than 50 (150) days in the water at the time of their interception. We note that the biofouling times adopted here ranged between 50 and 200 days.

We then calculated the total number of particles (expressed as a percentage of the total number

of particles released $N \simeq 1.472 \times 10^8$) intercepted by the first 10 crossroads as a function of the time spent in the water at the time of their interception (Fig. 14, lower panel) to determine if the ranking of the crossroads could be affected by the biofouling time. The number of particles intercepted did not change significantly for biofouling times larger than ~50–100 days; below that time period, only the ranking of few crossroads changed. These findings confirmed that the biofouling effect, even if not considered explicitly, did not affect significantly the crossroads distribution, as virtual particles were mainly intercepted during their first days in the water.

7 Sensitivity to shore steepness

In order to analyze the relative importance of shore steepness, we compared two scenarios: one included the shore steepness (Scenario SS) and one did not (Scenario NSS). In Scenario NSS, particles that reached land were automatically considered as beached. The standard methodology (M 1.2.3) was applied in Scenario SS to determine beaching. Both scenarios were run with the same K_h and T_W values (0 m^2/s and 50 days, respectively). Particle concentrations for the five Mediterranean sub-basins were calculated using the same method as for the other scenarios (M 1.2.8). These quantities were compared with the eight categories of *in situ* particle concentrations (Fig. 15, left panel). The correlation coefficients obtained with Scenario NSS were significant for Category 2 ($p<0.05$), and 3 and 4 ($p<0.01$). However, R^2 was always less than the corresponding value calculated with Scenario SS (Fig. 15, right panel). In addition, Scenario NSS displayed no significant correlation in Categories 7 and 8.

The simulated particle concentrations as a function of distance from shore (see Section S.2 for calculation details) were compared with the *in situ* observations (Categories 1 and 2). Lower correlation coefficients were obtained for Scenario NSS (Fig. 16, left panels) compared to those for Scenario SS (Fig. 16, right panels: Category 1, NSS R^2=0.79, SS R^2=0.96; Category 2, NSS R^2=0.84, SS R^2=0.97). In addition, *in situ* and simulated distributions were not similar ($p >$

0.05) for Scenario NSS, whereas they were for Scenario SS ($p < 0.05$).

These findings indicate that taking into account shore steepness improved the performance of the Track-MPD model, increasing the agreement with observations by improving the beaching description.

8 Sensitivity of surface sinking and net beaching rates on K_h and T_W

8.1 Horizontal diffusion K_h and half-life on the beach T_W

Supplementary Figure 17 shows the surface sinking and the net beaching rates for each of the 16 scenarios. In general, the two metrics did not change consistently across the different runs. Increasing the half-life of beached plastics T_W decreased slightly the number of beached particles and, in turn, increased the surface sinking rate. When increasing the horizontal diffusion K_h, the number of beached particles did not change significantly, while the surface sinking rate was smoothed.

8.2 Proportions of particles emitted from cities p_C, rivers p_R, and vessels p_V

The surface sinking and the net beaching rates were calculated using four different proportions of particles emitted from cities p_C, rivers p_R, and vessels p_V (upper table of Supplementary Fig. 5). The results (Fig. 19) show that the beaching rate did not changed significantly. The surface sinking rate was lower in Case 2 only, due to the lower input from vessels, but the pattern remained identical. This result is consistent with the fact that particles released from vessels spent more time in water, and contributed to almost 50% of the surface sinking rate (Supplementary Fig. 7, third and fourth panels). Overall, this sensitivity test shows the robustness of the surface sinking and net beaching rates with respect to the proportion of particles emitted from cities p_C,

rivers p_R, and vessels p_V.

9 Qualitative comparison of net beaching rates and observations of beached plastic items

Poeta et al., 2016 (*9*) reported seasonal variations in plastic deposition on four beaches close to Montalto (central Italy) between spring 2014 and winter 2015. Supplementary Figure 18 shows: (i) the mean values obtained by the authors (*9*); and (ii) the seasonal net beaching rate predicted by the TrackMPD model for the same area (note that both quantities were normalized to allow comparison). A good qualitative agreement emerged between the two metrics, except for winter, during which the difference was more pronounced. These results are evidence that the TrackMPD model provides good estimates of beaching rates at local and seasonal scales, although further improvements are necessary. In addition, the net beaching rates calculated here agree qualitatively with the findings of (*10*) and (*11*). The two studies observed massive beach pollution in the Cilician basin, which our model predicts to be significanlty affected by net beaching rates ($\sim$10–14 kg/km/day).

10 Qualitative comparison of surface sinking rates and observations of seafloor plastic concentration

Supplementary Table 1 reports the literature on seafloor plastic surveys at several locations across the Mediterranean basin. Before comparing them with the surface sinking rates predicted by our model, we stress that the difficulty of this task was exacerbated by several factors. Different sampling techniques were adopted in the seafloor plastic observations (for instance, bottom trawls, which can be conducted only on muddy bottoms, or scuba-dive samplings). The minimum size of the plastic debris measured varied from study to study. In addition, plastic

concentration was measured either as items/km^2, as kg/km^2 or as items per kg of dry sediment. The debris collected were composed of plastic with a density larger than seawater, which were not modelled in the present study. Finally, campaigns were conducted on different years, and this could obviously lead to important differences in the plastic concentrations measured.

Despite these premises, the highest concentrations of benthic plastics in the Mediterranean to date were in Mersin bay (*12*) and in the Gulf of Antalya (*13*), both of them in the Cilician basin. Consistent with these observations, our modeling results suggested that these regions experienced the highest surface sinking rates in the Mediterranean (Figs. 4 and S17). One of the lowest seafloor values recorded in the Mediterranean was on the Sardinian shelf (*14*), which was also one of the regions with the lowest surface sinking rates in our model. The study of (*15*) in the Gulf of Lion describes a rather stable-over-time region of enhanced bottom plastic concentrations around 5°E. This area was characterized by surface sinking rates which were larger than in the rest of the Gulf of Lion.

Supplementary Figure 1

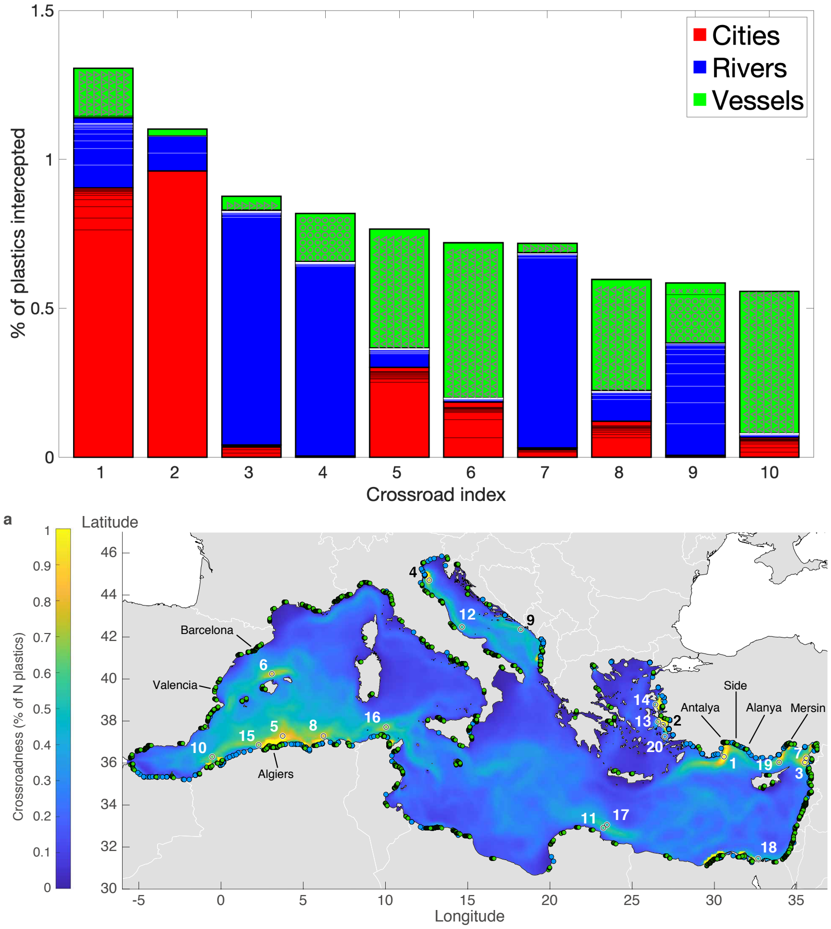

Figure 1: Scenario M. **First panel:** Each column shows the percentage (y-axis) of virtual particles (relative to the total number of particles released $N \simeq 1.472 \times 10^8$) intercepted by each of the first 10 crossroads. The colors of the 3 rectangles forming each column indicate the origin of the particles: red for particles released from cities, blue for rivers, and green for vessels. The black and white lines inside the red and blue rectangles split the contribution of different cities and rivers, respectively. For instance, the first crossroad intercepted $\sim$0.75% of particles coming from a single city (Antalya, Turkey), and $\sim$0.10% from three other cities: Mersin (0.04%); Alanya (0.04%); and Side (0.02%). The shapes of the symbols inside the green rectangles indicate the basin in which the particles were released: left-pointing triangles, western Mediterranean; dots, Tyrrhenian Sea; open circles, Adriatic Sea; crosses, Aegean Sea; downward triangles, central Mediterranean; right-pointing triangles, eastern basin. To facilitate the reading, the **second panel** shows the crossroadness and the 20 most important crossroads for Scenario M, as reported in Fig. 3.

Supplementary Figure 2

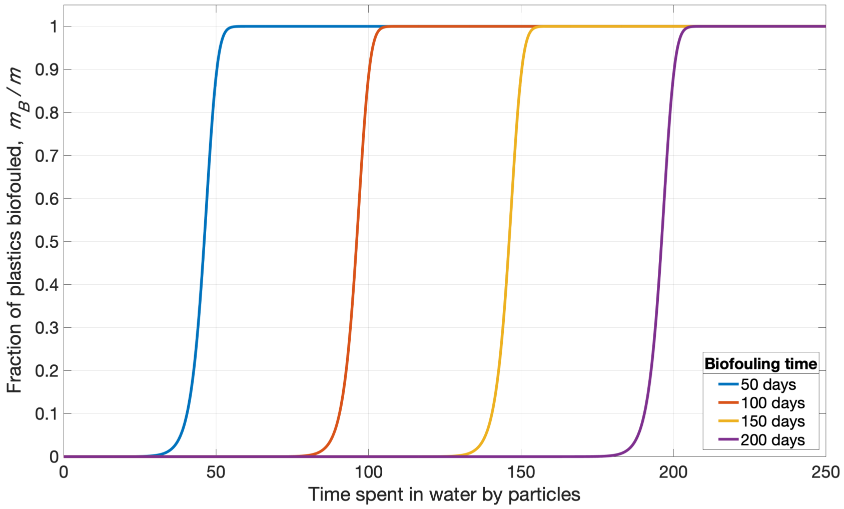

Figure 2: Fraction of plastics mass which is biofouled (m_B/m) as a function of the time spent in water, calculated using Expr. (2) (M 3.2.5). The curves are calculated considering four different biofouling times T_{BF}: 50, 100, 150, and 200 days.

Supplementary Figure 3

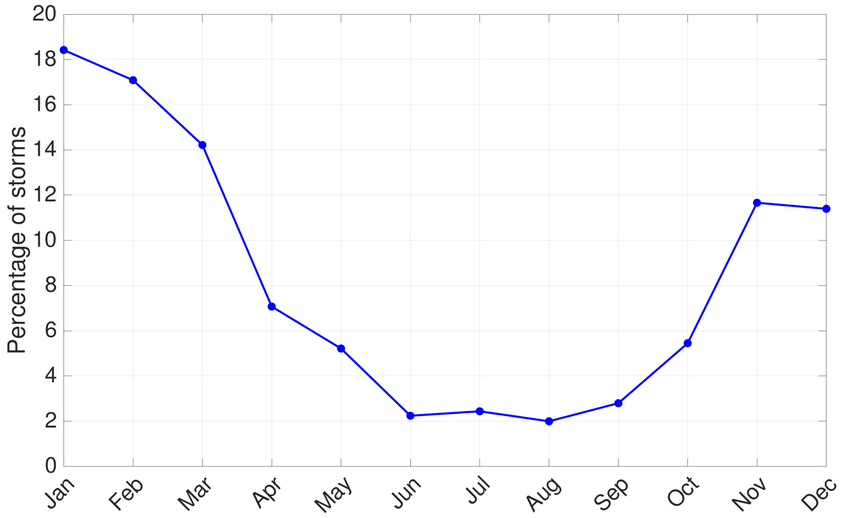

Figure 3: Number of storms (expressed as percentage) in the Mediterranean coasts during the simulation period (2013–2017). The storms were detected from sea wave height time series and used to identify washing-off events (M 3.2.4)

Supplementary Figure 4

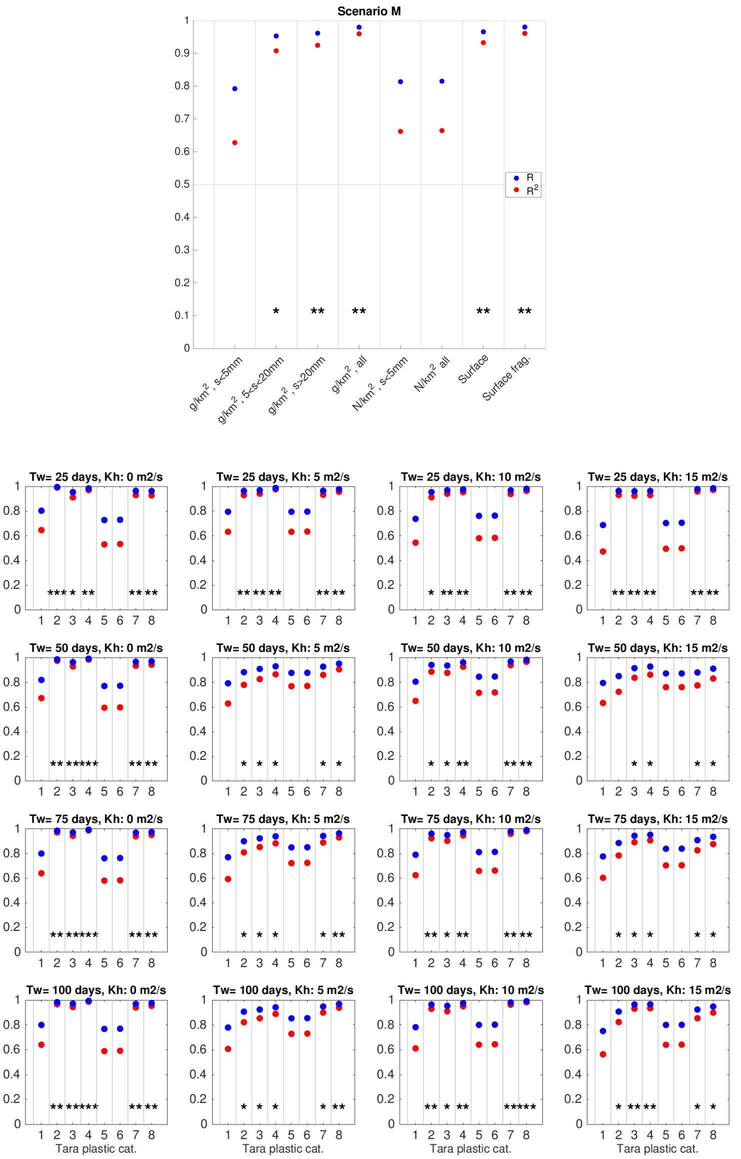

Figure 4: **First panel:** Sub-basin comparison of virtual particle concentrations (Scenario M) with the 8 *in situ* categories of observed plastic concentration (x-axis). Blue and red dots indicate R and R^2 coefficient values, respectively. Black stars, when present, identify a significant correlation between simulated and *in situ* concentrations (*: p<0.05; **: p<0.01; ***: p<0.001). **Second panel:** The same comparison, but for each of the 16 scenarios separately. The set of K_h and T_W values used is shown in the title of each subplot.

Supplementary Figure 5

	p_c (%)	p_r (%)	p_v (%)
Case 1	40	40	20
Case 2	62	32	6
Case 3	60	20	20
Case 4	60	25	15

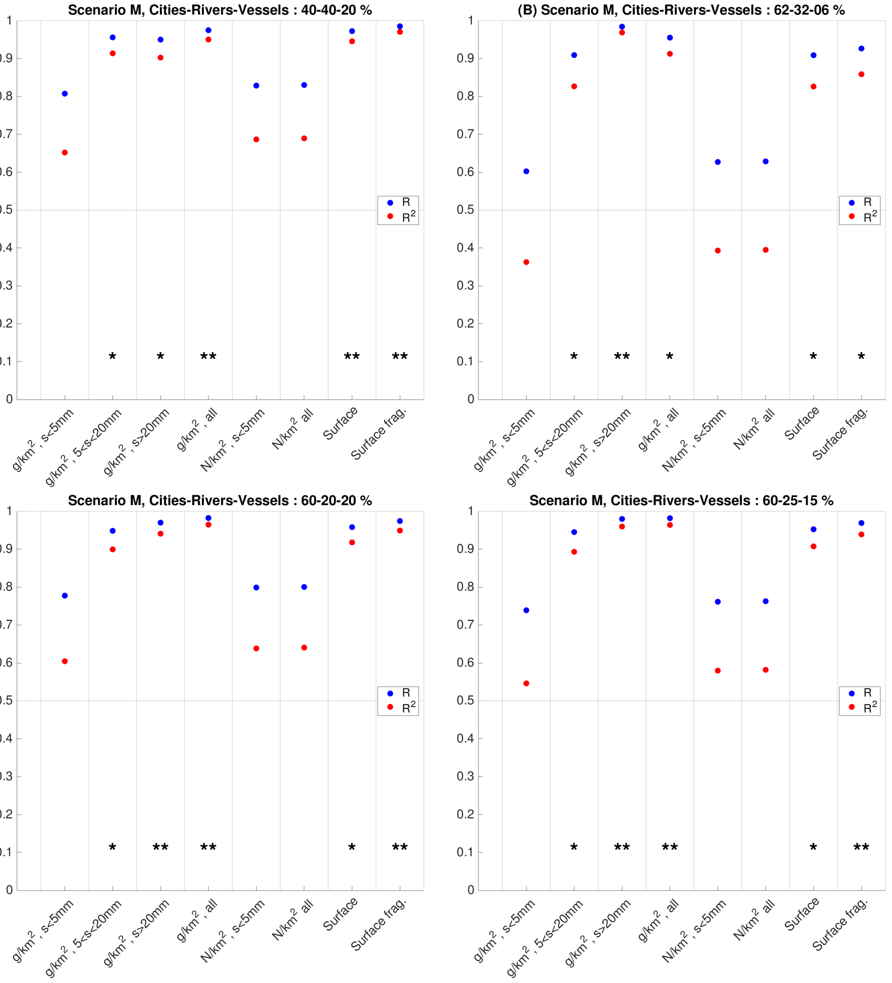

Figure 5: **Upper table**: list of the proportion of particles released from cities, rivers, and vessels (p_C, p_R, and p_V) used to test the sensitivity of the results with respect to these parameters. **Panels A–D**: sub-basin comparison of virtual particle concentrations (Scenario M) with the 8 *in situ* categories of observed plastic concentration (x-axis). The R and R^2 cales (y-axis) were calculated as in the first panel of Supplementary Fig. 4, for different values of p_C, p_R, and p_V (case 1–4, upper table).

Supplementary Figure 6

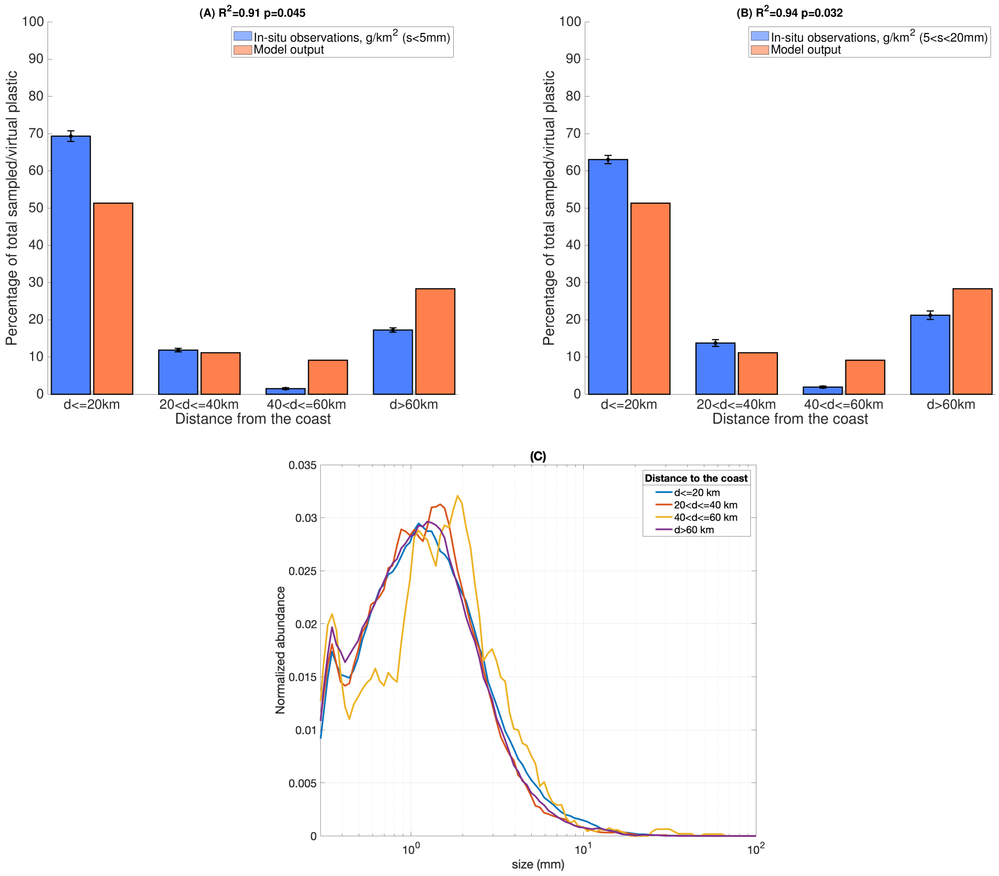

Figure 6: Distribution of *in situ* (blue columns) and virtual (orange columns) particle concentrations at the location of the Tara Expedition stations as functions of the distance from the shore. Virtual particle concentrations were obtained for Scenario M. *In situ* concentrations refer to Categories 1 (**panel A**) and 2 (**panel B**) and are showed with relative uncertainties (standard deviation: black error bars). Both concentrations (simulated and *in situ*) were normalized to allow comparison. R^2 values for the correlation between the two distributions (Pearson test) were 0.91 and 0.94, respectively ($p<0.05$ in both cases). **Panel C**: normalised particle size distribution according to the distance from the shore.

Supplementary Figure 7

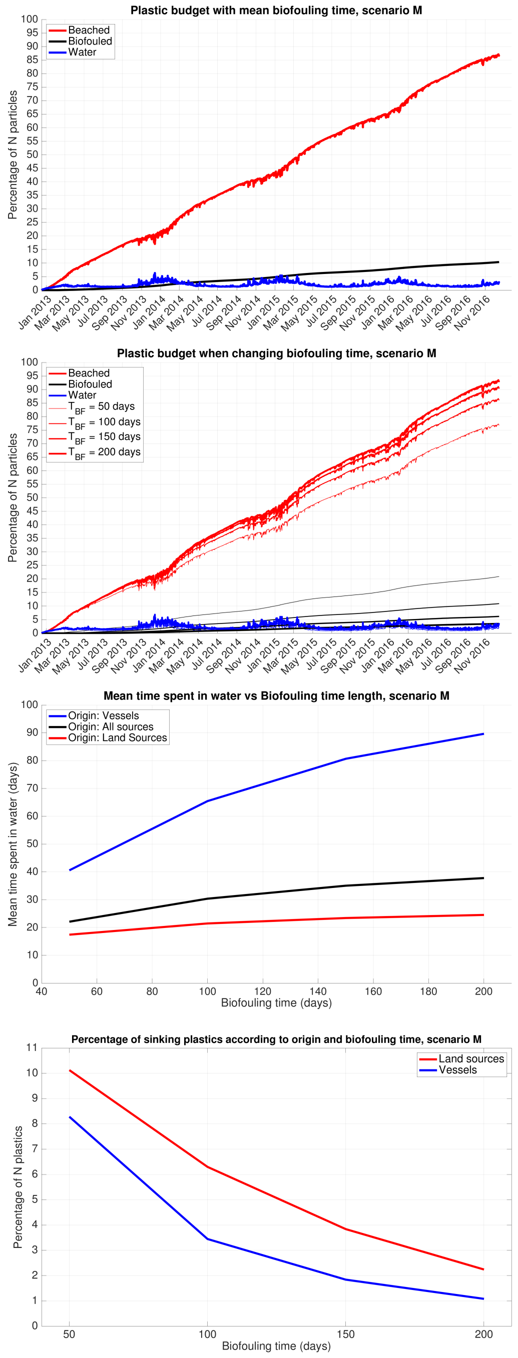

Figure 7: (on previous page) **First panel:** Percentage of the total number N of virtual particles released that, during the release period (January 1, 2013 to December 31, 2016; x-axis), beached (red line), sank (black line), or remained floating (blue line). The three quantities were obtained from the ensemble average of simulations with four different biofouling times T_{BF} (50, 100, 150, and 200 days). These are shown separately in the **second panel**. Lines of the same thickness refer to the values computed with the same biofouling time: thinnest lines T_{BF}=50 days, thickest line T_{BF}=200 days; intermediate thicknesses T_{BF}=100 and T_{BF}=150 days. **Third panel:** Mean time (in days, y-axis) that particles spent in the water as a function of the biofouling time T_{BF} (in days, x-axis). Black line: all particles; red line: particles released from land sources (cities and rivers); blue line: particles released from vessels. **Fourth panel:** Percentage of the N virtual particles (y-axis) that sank due to biofouling as a function of the biofouling time T_{BF} (x-axis). Red line: particles released from land sources; blue line: particles released from vessels.

Supplementary Figure 8

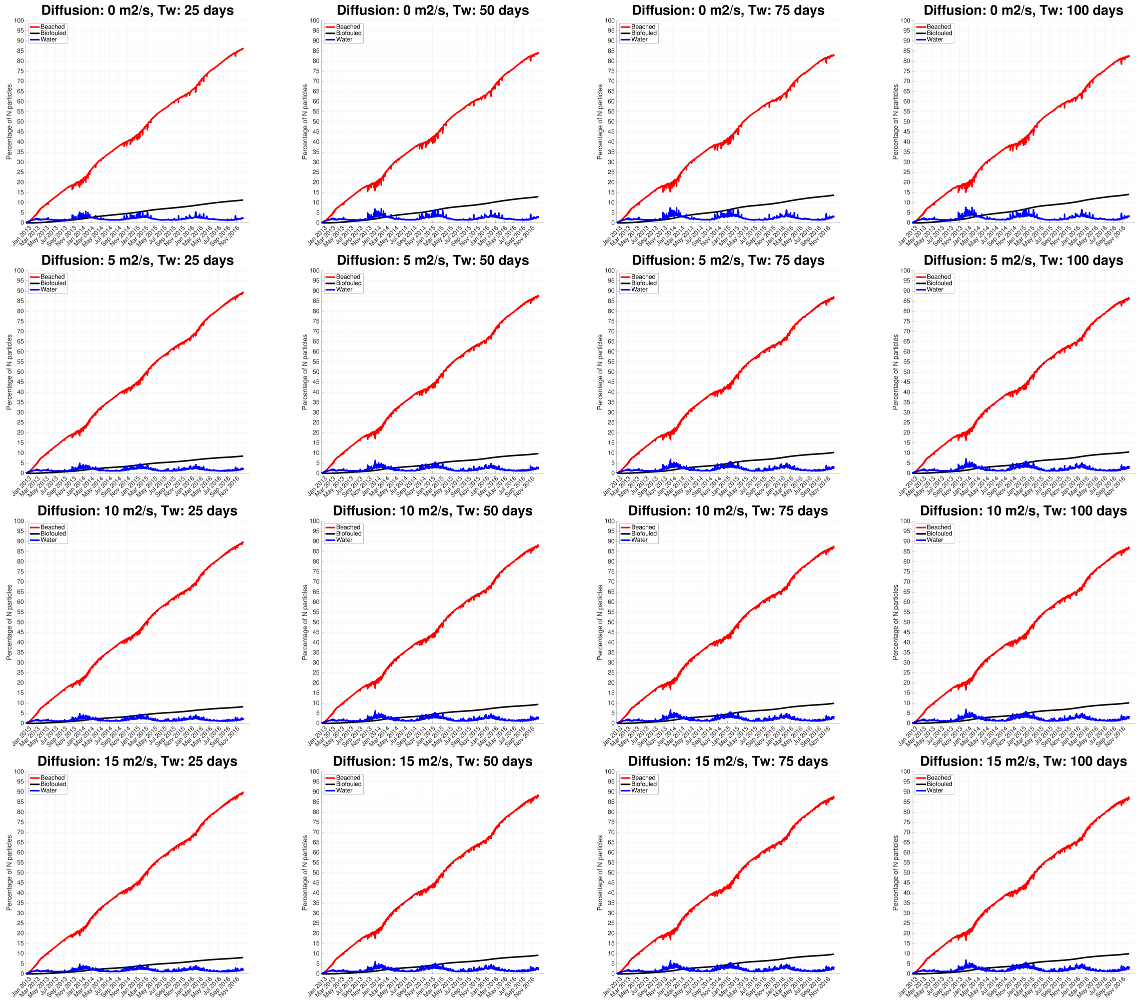

Figure 8: Plastic budget, calculated as in the first panel of Supplementary Fig. 7, for each of the 16 scenarios separately. The K_h and T_W values used are shown in the title of each subplot.

Supplementary Figure 9

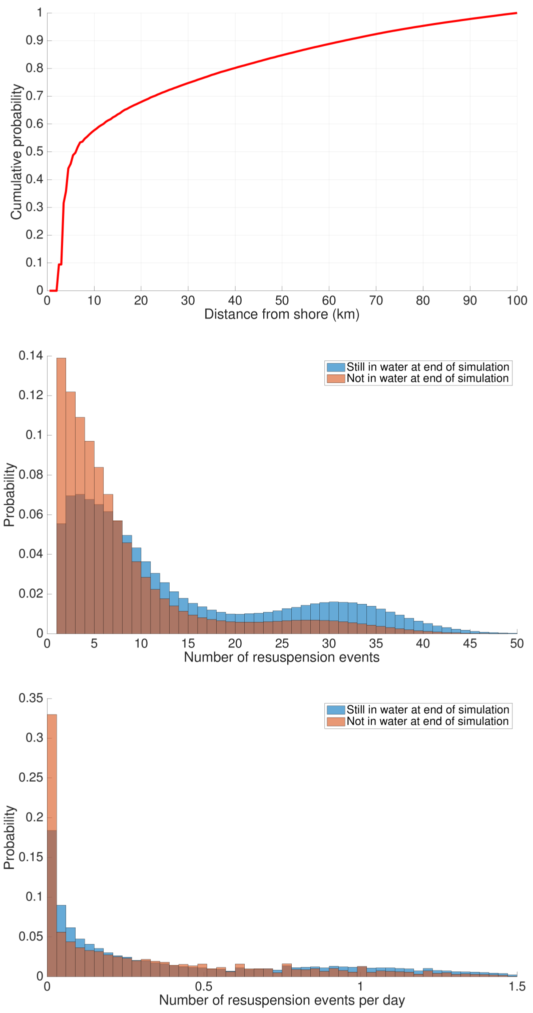

Figure 9: **First panel:** Cumulative pdf of distances from shore of particles that were still floating in the water at the end of the advective time. **Second panel:** pdf of number of washing-off events for particles (i) still in the water (blue columns) and (ii) not in the water (in that they were either beached or biofouled; orange columns) at the end of the advective time. The color of the columns where the two distributions superpose is brown. **Third panel**: pdf of the frequency of washing-off events per day of particles (i) still in the water (blue columns) and (ii) not in the water (orange columns). The frequency was obtained by dividing the number of washing-off events of each particle by the time it spent in the water.

Supplementary Figure 10

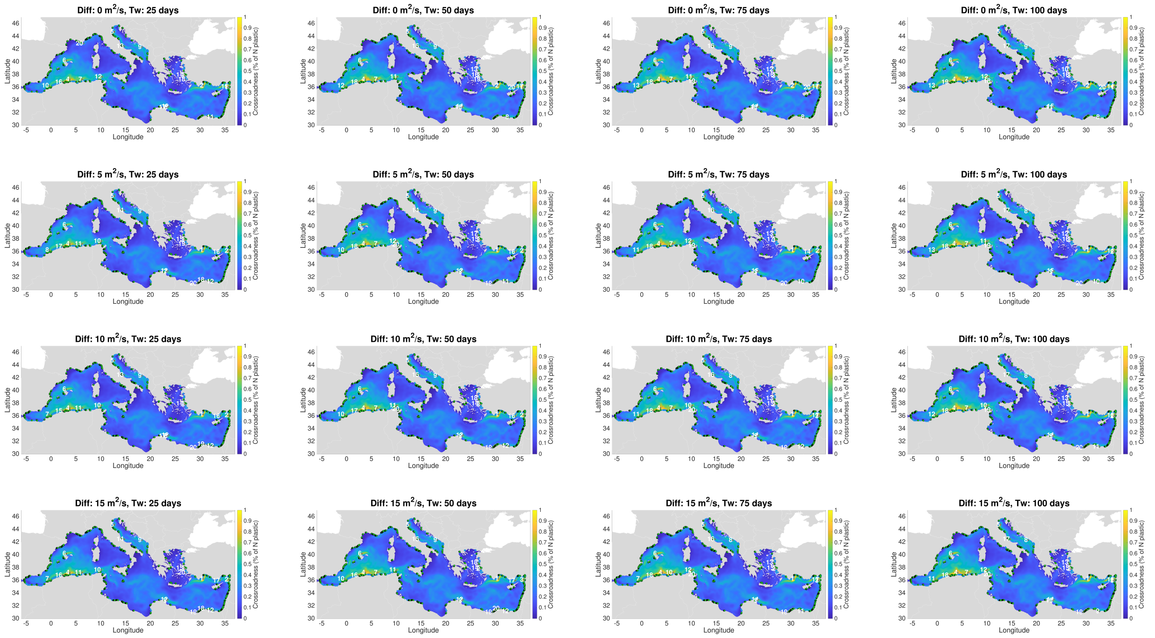

Figure 10: Plastic crossroadness and the 20 most important crossroads computed for each of the 16 scenarios separately, as in Fig. 3. $\sigma = 0.1°$. The K_h and T_W values used are shown in the title of each subplot.

Supplementary Figure 11

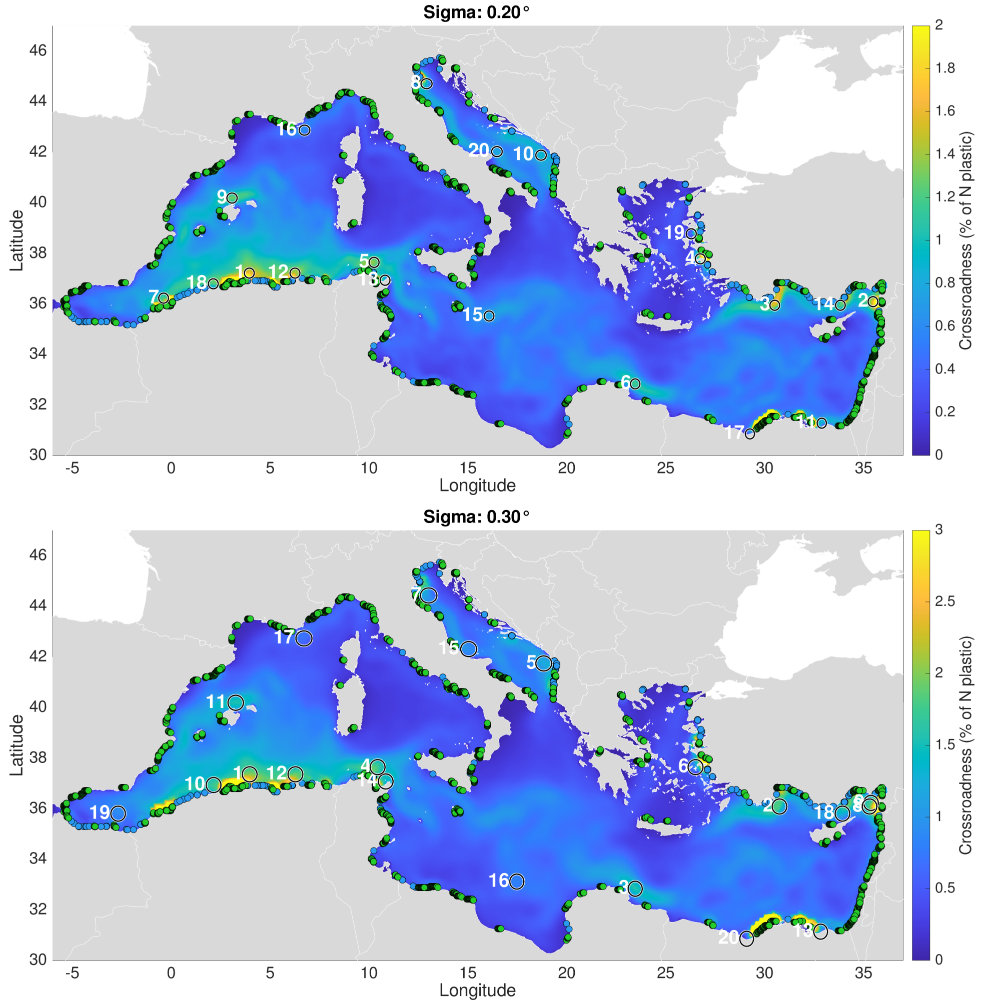

Figure 11: Plastic crossroadness and 20 most important crossroads calculated with two different σ values (**first panel:** $\sigma = 0.2°$; **second panel:** $\sigma = 0.3°$) for Scenario M. Method as in Fig. 3.

Supplementary Figure 12

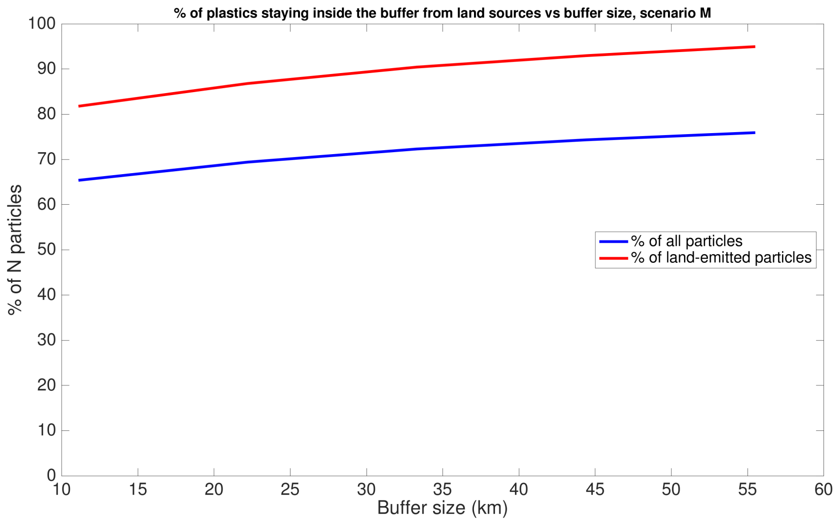

Figure 12: Scenario M. Blue line: percentage of the N virtual particles that remained inside the buffer region around each land source as a function of the buffer size (x-axis). Red line: corresponding percentage of the total number of virtual particles released from land sources.

Supplementary Figure 13

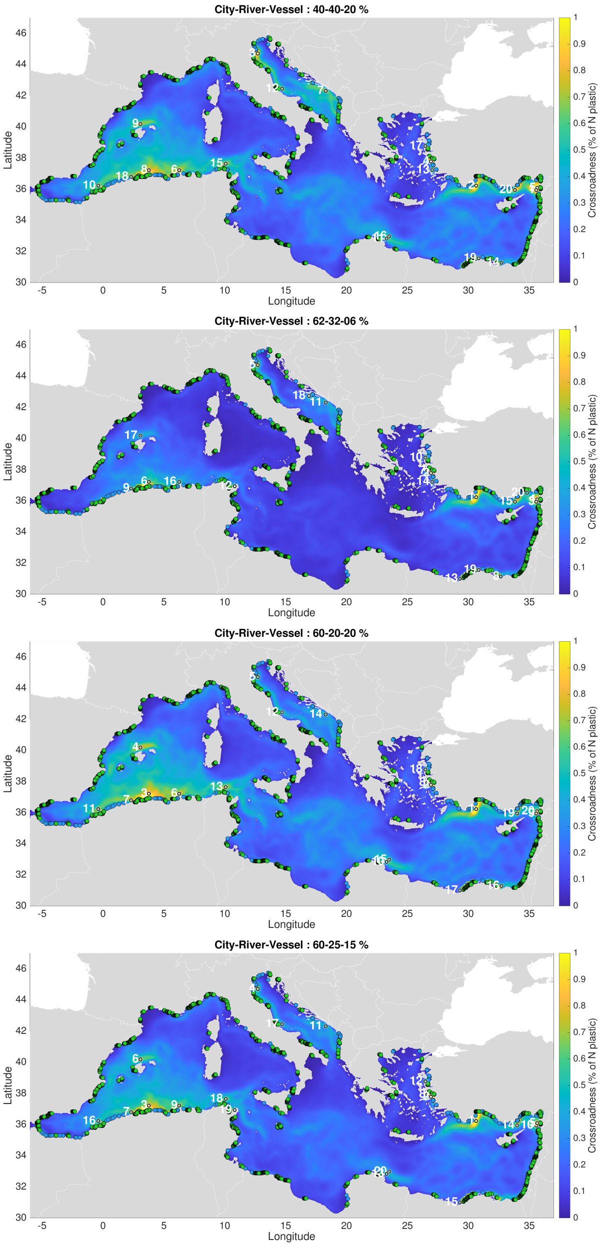

Figure 13: Plastic crossroadness and 20 most important crossroads calculated using different proportion of particles released from cities, rivers, and vessels (p_C, p_R, and p_V; upper table in Supplementary Fig. 5).

Supplementary Figure 14

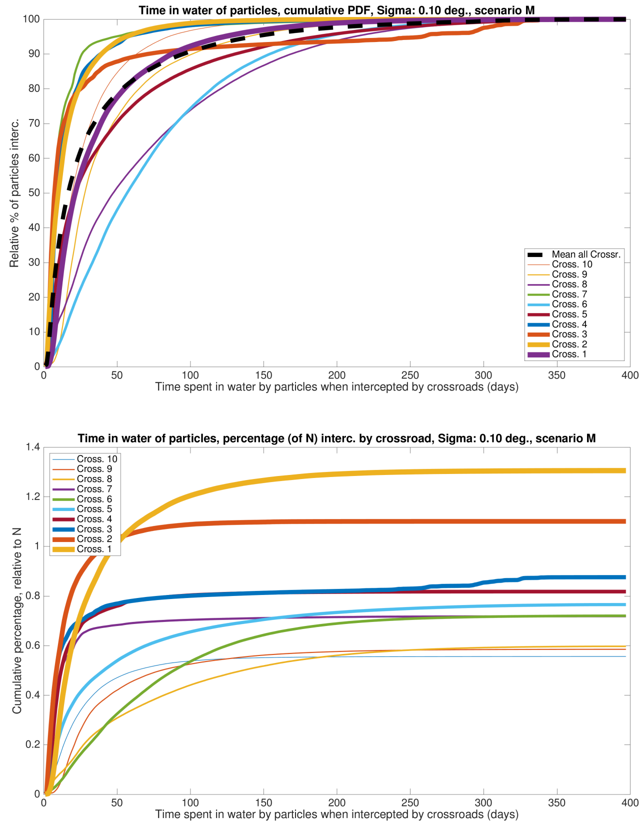

Figure 14: Scenario M. **First panel:** Cumulative pdf of the time spent in the water by the virtual particles from their release until they were intercepted by a crossroad ($\sigma = 0.1°$). Solid lines: cumulative pdfs calculated by considering all the particles intercepted by one of the first ten plastic crossroads; black dotted line: by considering all the virtual particles intercepted by all the crossroads. For example $\sim$80% of the virtual particles, at the moment at which they were intercepted, had spent less than 50 days in the water since their release. **Second panel:** As for the first panel but with the percentage relative to the total number of particles released $N \simeq 1.472 \times 10^8$.

Supplementary Figure 15

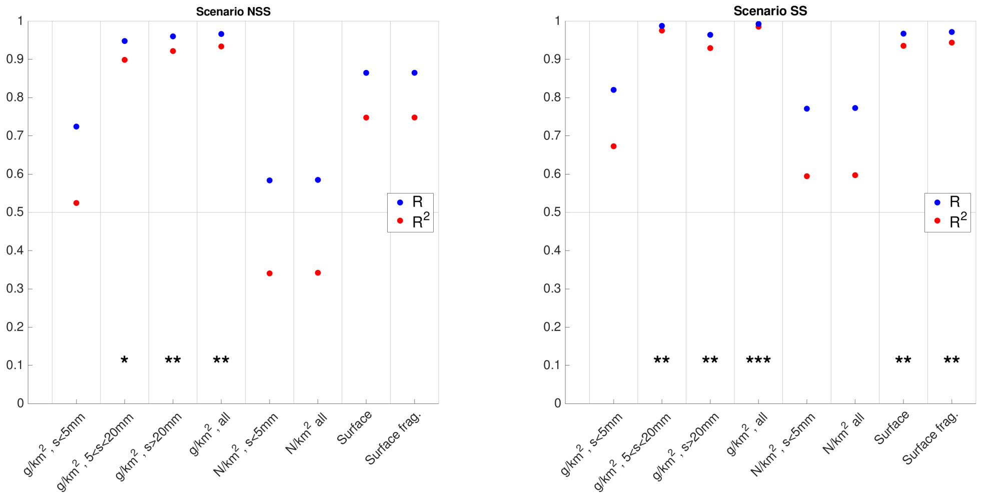

Figure 15: Comparison of simulated and *in situ* sub-basin particle concentrations calculated as in Fig. 4. **Left panel:** correlations for Scenario NSS, in which shore steepness was not taken into account. **Right panel:** correlations for the same scenario but with shore steepness taken into account (Scenario SS).

Supplementary Figure 16

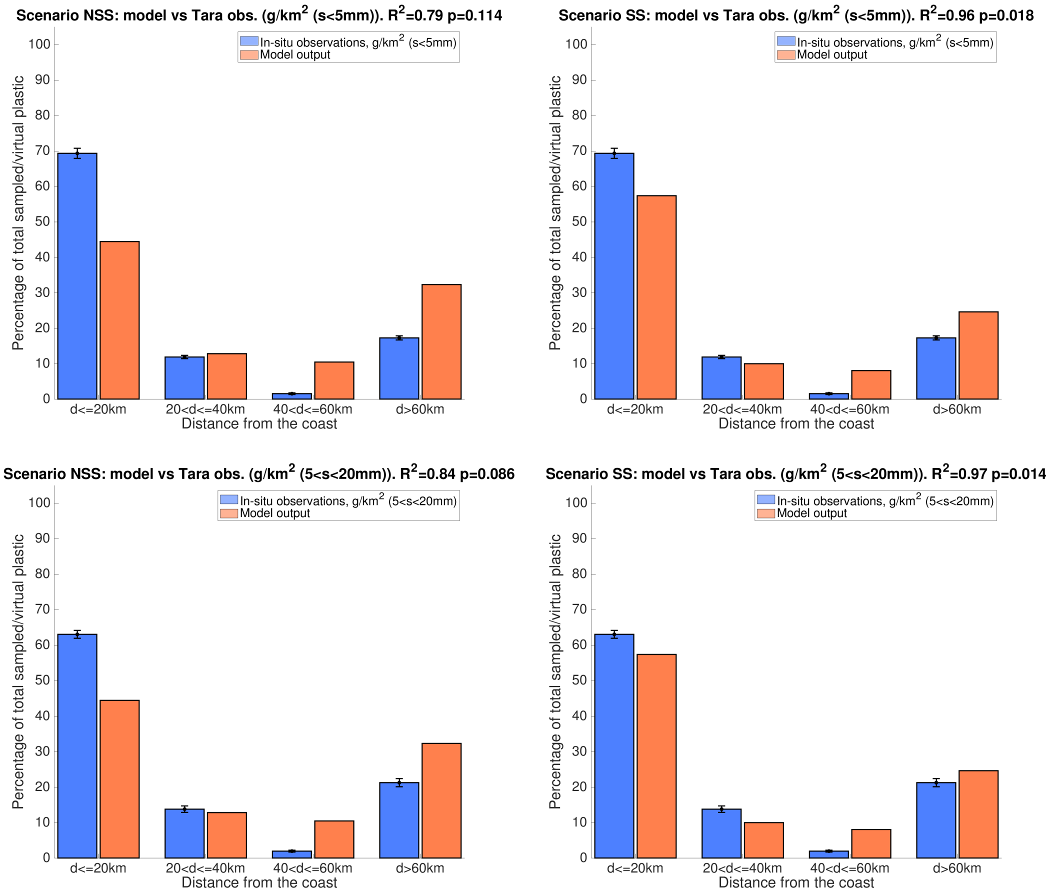

Figure 16: Comparison of *in situ* (blue columns) and virtual (orange columns) normalized particle concentrations as a function of distance from the shore (calculated as in Fig. 6). *In situ* particle concentrations refer to Category 1 (**first row**) and 2 (**second row**) and are showed with relative uncertainties (standard deviation: black error bars). Virtual particle concentrations were calculated without taking into account shore steepness (**left panels**, Scenario NSS) and with taking it into account (**right panels**, Scenario SS). R^2=0.79 (upper left panel) and 0.84 (lower left) for Scenario NSS (both p-values >0.05); 0.96 (upper right panel) and 0.97 (lower right) for Scenario SS (both p-values <0.05).

Supplementary Figure 17

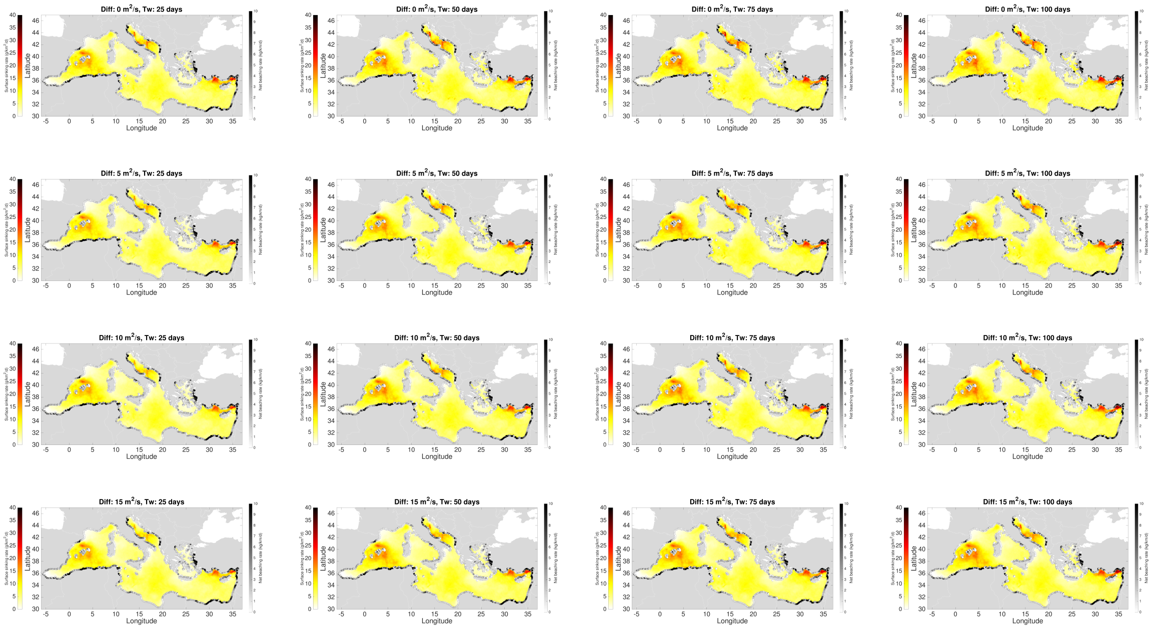

Figure 17: Surface sinking and net beaching rates, calculated as in Fig. 4, for each of the 16 scenarios separately. The K_h and T_W values used are shown in the title of each subplot.

Supplementary Figure 18

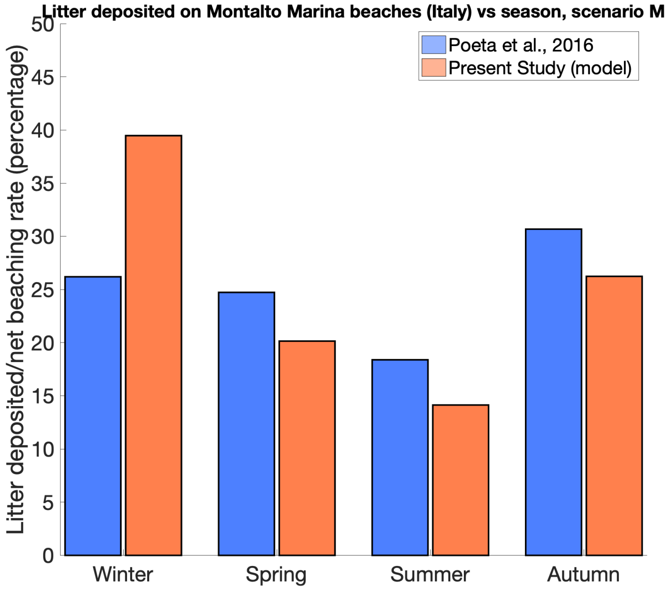

Figure 18: **Blue columns:** seasonal deposition of plastic litter on Montalto beaches (west coast of central Italy), measured between spring 2014 and winter 2015 (*9*). **Orange columns:** simulated seasonal net beaching rate on the same beaches. Both quantities were normalized so that the sum over the four seasons was equal to 1.

Supplementary Figure 19

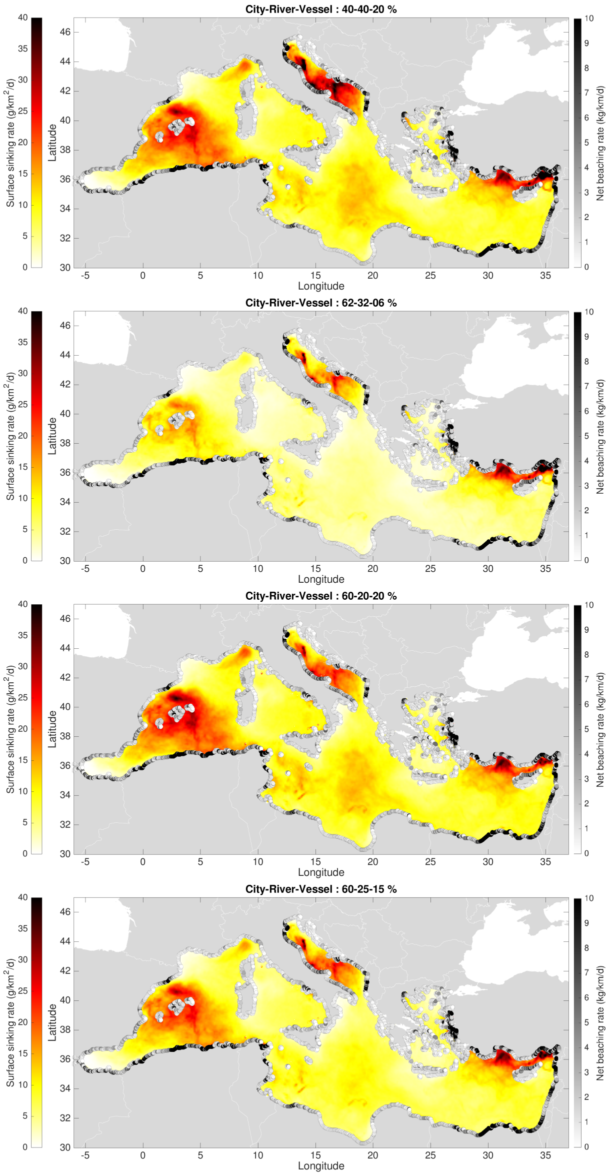

Figure 19: Surface sinking and net beaching rates, calculated as in Fig. 4, but using different proportion of particles released from cities, rivers, and vessels (p_C, p_R, and p_V; upper table in Supplementary Fig. 5).

Supplementary Table 1

Reference	Items/km2	Kg/km2	Items/(kg of dry sediment)	Sampling year	Sampling location	Sampling depth (m)	Mesh size	Notes
Galgani et al., 2000	92	/	/	1994-1997	Gulf of Lion	50-1000	> 1 cm	
	105	/	/	1998	East Corsica		> 2 cm	
	263	/	/	1998	Adriatic Sea		> 2 cm	
Stefatos et al., 1999	199	/	/	Nov 1997	Patras Gulf	80-120	> 1.5 cm	
	70	/	/	May 1998	Echinades Gulf	247-360		
Koutsodendris et al., 2008	142	/	/	2000-2003	Patras and Lakonikos Gulfs	15-320	> 1.5 cm	Small gulfs, complex topography
Ioakeimidis et al., 2014	1211	/	/	Jan-Mar 2013	Saronikos Gulf	50-450	> 5 cm	Small gulfs, complex topography
	611	/	/		Patras Gulf			
	416	/	/		Echinades Gulf			
	24	/	/		South of Cyprus			
Romeo et al., 2015	/	/	2±2	Unspecified	Malta Grand Harbour	4-22	less than 5mm	Sampling inside the port
Melli et al., 2017	11715	/	/	May-Jun 2014 and Jul 2015	North of Po delta	21-23	Unspecified (video assessment)	
Gundogdu et al., 2017	2670	86.3	/	Unspecified	Mersin Bay, Cilician basin	Unspecified	Unspecified	
Olguner et al., 2018	10-470	137.25	/	Oct 2014-Feb 2015	Antalya bay	10-300	> 2.4 cm	
García-Rivera et al., 2017	/	0-11	/	Feb-Aug 2014	0 to 35 km from Alicante	50-700	> 4/5 cm	
Galimany et al., 2019	/	4.5-52.4	/	2016-2017	West of Barcelona	5-70	> 4 cm	
Consoli et al., 2018	803	/	/	Jun-Jul 2012	South of Sicily	5-30	Unspecified (video assessment)	
Alvito et al., 2018	35 ± 4	7.35 ± 2.4	/	2013-2015	Sardinia	0-800	>2 cm	
Loulad et al., 2019	/	25 ± 60	/	2012-2015	Morocco	10-600	Unspecified	
Pasternak et al., 2019	30319	/	/	2012-2015	Israel	< 3	Unspecified (observ. through scuba-dives	Observ. very close to the shore
	6745	/	/			0-5		

Table 1: Literature on Mediterranean seafloor plastic concentration.

Supplementary References

1. L.-M. Lebreton, S. Greer, J. Borrero, *Marine Pollution Bulletin* **64**, 653 (2012).
2. M. L. A. Kaandorp, H. A. Dijkstra, E. van Sebille, *Environmental Science & Technology* **54**, 11980 (2020). PMID: 32852202.
3. M. L. Pedrotti, *et al.*, *PLOS ONE* **11**, 1 (2016).
4. S. Liubartseva, G. Coppini, R. Lecci, E. Clementi, *Marine Pollution Bulletin* **129**, 151 (2018).
5. L. Lebreton, M. Egger, B. Slat, *Scientific Reports* **9**, 12922 (2019).
6. J. Mansui, A. Molcard, Y. Ourmires, *Marine Pollution Bulletin* **91**, 249 (2015).
7. F. Guerrini, L. Mari, R. Casagrandi, *Frontiers in Marine Science* **6**, 299 (2019).
8. J. Mansui, *et al.*, *Progress in Oceanography* **182**, 102268 (2020).
9. G. Poeta, C. Battisti, M. Bazzichetto, A. T. Acosta, *Marine Pollution Bulletin* **113**, 266 (2016).
10. C. Aydın, O. Güven, B. Salihoğlu, A. E. Kıdeyş, *Turkish Journal of Fisheries and Aquatic Sciences* **16**, 029 (2016).
11. S. Gündoğdu, C. Çevik, *Environmental Pollution* **255**, 113351 (2019).
12. S. Gündoğdu, C. Çevik, S. Karaca, *Marine Pollution Bulletin* **124**, 147 (2017).
13. M. T. Olguner, C. Olguner, E. Mutlu, M. C. Deval, *Marine Pollution Bulletin* **136**, 171 (2018).

14. A. Alvito, *et al.*, *Waste Management* **75**, 131 (2018).

15. F. Galgani, *et al.*, *Marine Pollution Bulletin* **40**, 516 (2000).

16. A. Stefatos, M. Charalampakis, G. Papatheodorou, G. Ferentinos, *Marine Pollution Bulletin* **38**, 389 (1999).

17. A. Koutsodendris, G. Papatheodorou, O. Kougiourouki, M. Georgiadis, *Estuarine, Coastal and Shelf Science* **77**, 501 (2008).

18. C. Ioakeimidis, *et al.*, *Marine Pollution Bulletin* **89**, 296 (2014).

19. T. Romeo, *et al.*, *Environmental Monitoring and Assessment* **187**, 747 (2015).

20. V. Melli, *et al.*, *Marine Pollution Bulletin* **114**, 821 (2017).

21. S. García-Rivera, J. L. S. Lizaso, J. M. B. Millán, *Marine Pollution Bulletin* **121**, 249 (2017).

22. E. Galimany, *et al.*, *Waste Management* **95**, 620 (2019).

23. P. Consoli, *et al.*, *Marine Pollution Bulletin* **136**, 243 (2018).

24. S. Loulad, R. Houssa, N. E. Ouamari, H. Rhinane, *Marine Pollution Bulletin* **139**, 163 (2019).

25. G. Pasternak, *et al.*, *Ocean & Coastal Management* **175**, 17 (2019).
